# Sharing of Clinical Trial Data and Samples: The Cancer Patient Perspective

**DOI:** 10.3389/fmed.2020.00033

**Published:** 2020-02-11

**Authors:** Stefanie Broes, Ciska Verbaanderd, Minne Casteels, Denis Lacombe, Isabelle Huys

**Affiliations:** ^1^Clinical Pharmacology and Pharmacotherapy, Department of Pharmaceutical and Pharmacological Sciences, KU Leuven, Leuven, Belgium; ^2^European Organisation for Research and Treatment of Cancer, Brussels, Belgium; ^3^Anticancer Fund, Strombeek-Bever, Belgium

**Keywords:** data sharing, sample sharing, patient perspective, ethical and legal implications, neoplasms, e-consent

## Abstract

**Introduction:** Today, many initiatives and papers are devoted to clinical trial data (and to a lesser extent sample) sharing. Journal editors, pharmaceutical companies, funding agencies, governmental organizations, regulators, and clinical investigators have been debating the legal, ethical, and social implications of clinical data and sample sharing for several years. However, only little research has been conducted to unveil the patient perspective.

**Aim:** To substantiate the current debate, we aimed to explore the attitudes of patients toward the re-use of clinical trial samples and data and to determine how they would prefer to be involved in this process.

**Materials and Methods:** Sixteen in-depth interviews were conducted with cancer patients currently participating in a clinical trial.

**Results:** This study indicates a general willingness of cancer patients participating in a clinical trial to allow re-use of their clinical trial data and/or samples by the original research team, and a generally open approach to share data and/or samples with other research teams, but some would like to be informed in this case. Despite divergent opinions about how patients prefer to be engaged, ranging from passive donors up to those explicitly wanting more control, participants expressed positive opinions toward technical solutions that allow indicating their preferences.

**Conclusion:** Patients were open to sharing and re-use of data and samples to advance medical research but opinions varied on the level of patient involvement and the need for re-consent. A stratified approach for consent that allows individualization of data and sample sharing preferences may be useful, yet the implementation of such an approach warrants further research.

## Introduction

Interventional clinical research leads to a change in the clinical management of the patient (e.g., by the experimental intervention). Therefore, informing patients about the nature, significance, implications, and risks of the research they will participate in and obtaining their subsequent consent are established procedures embedded in current ethical and legal frameworks [i.e., Declaration of Helsinki (1), EU Clinical Trial Directive (2), and upcoming Clinical Trial Regulation (3)]. In addition, most ethical frameworks stipulate–in line with the data protection and biobanking legislative frameworks–that it is required to re-inform people about the (further) processing of their personal data and human samples, unless impossible to do so (1). Even though the mere further processing of data or samples (“secondary use”) does not lead to a new intervention, it may still lead to discussions about ethical and moral values, for instance where patients have not consented or have not been informed about such further use (4). Respecting one's consent is important since trust in the participant-researcher relationship is maintained insofar as there is proper use of the donated items in accordance with what was agreed (4).

Privacy consequences in case of data breach can be substantial. Disclosure of sensitive, personal data may lead to embarrassment, stigmatization, discrimination for loans or insurances, unwillingly unveiling biological ties, loss of employment, etc. In this respect, anonymizing the data may be a welcomed solution, since this is not subject to legal EU data protection requirements (5, 6). However, anonymizing data is not always advisable, desirable or even possible and even if data are anonymized at one point in time, safeguarding against re-identification can be challenging. For example, Lin Z and colleagues demonstrated that as little as 30–80 single nucleotide polymorphisms (SNPs) from a single person can uniquely identify that person (7), and other examples exist (8, 9). Such examples stir up social concerns and can potentially undermine research participants' trust in research. Public concerns are further fueled by the extraordinary pace of technological developments and public communications about potential misuse of medical data, for instance by pharmaceutical companies (10, 11).

At present, the many initiatives and papers devoted to the topic of clinical trial data (and to a lesser extent sample) sharing illustrate the increasing attention that is being paid to this subject (12, 13). In a previous study, we identified the pros and cons of increased clinical trial data and sample sharing (14). The legal, ethical, and social implications of clinical data and sample sharing are largely being debated by journal editors, pharmaceutical companies, funding agencies, governmental organization, regulators, clinical investigators, etc. (15–20). Many uphold a moral obligation vis-à-vis study participants (i.e., “research participants *want* their data to be used for further research”) as the number one motivation for increased sharing efforts. Yet, it is unclear how the assumptions drawn by these stakeholders reflect the views of research participants, as only little empirical evidence is available when it comes to patient and research participant perspectives on the sharing of clinical trial research data and human samples. Moreover, only a small body of evidence is available on new tools to give patients a voice to express their opinion, and contribute to a transparent system where data are shared and re-used in accordance with the donors' preferences (21).

Some evidence exists from patient preference studies about the access and sharing of medical data captured in electronic health records (EHRs) (22, 23). Although not completely similar to the re-use of clinical trial data (and samples), since in the context of re-use of EHRs for research it constitutes a situation of re-purposing (i.e., from care to research) and data are not collected on the basis of informed consent [but on the basis of art. 8(3) EU Data Protection Directive (6), namely for the purposes of preventive medicine, medical diagnosis, the provision of care or treatment or the management of health-care services], interesting parallels can be drawn. A systematic literature review on this topic shows that the public has little knowledge on how their EHRs are shared and used for research purposes, and that a lack of transparency and engagement can undermine public trust (24). Furthermore, focus group participants expressed concerns about data sharing for commercial gain and the potential misuse of information (24). In view of these concerns, people may be more willing to share their medical data for research by public organizations (24). However, the United Kingdom government's care.data initiative, a program that enabled sharing anonymized EHRs with researchers outside the National Health Service (NHS), received widespread criticism and was stopped eventually in 2016 due to a lack of public trust (25). In addition, a survey with 1,011 respondents from 2014 indicated that a majority of the U.S. public had little trust in an integrated health data sharing system (26).

A patient at the European Patients' Forum stated the following: “*We, as patients, are increasingly aware of the value and importance of sharing our data. From the patients' perspective, use of health and genetic data is vital to advancing health research”* (27). At the risk of singling out opinions from (potentially) active and engaged patients, additional research is needed to understand the patient perspective on data and sample sharing. In 2016, Jones et al. conducted a survey on the topic of clinical trial data sharing with 799 (general) patients who entered the emergency department in a United States (US) hospital (28). Of these patients, 16% had previously participated in a trial. Eighty-five percent of the total group strongly favored clinical trial data sharing, and only 9% were against or strongly against it. Further, they report that approximately 85% of the survey respondents indicate that upfront disclosing a fully detailed data sharing plan is important since it increases transparency. These results provide guidance. However, the “patient” group was not specifically targeted toward clinical trial participants; but rather represents a broad category of people, which may obfuscate certain patient-specific attitudes. For the purpose of this study, we focused on a patient population participating in a trial in a particular domain, namely cancer. Further, we diversified between “re-use by the original research team” and “re-use by a new research team.” We propose that this might influence patients' viewpoint since they originally consented to use by one research team in specific, and not yet to an unknown group. We also inquired whether patients' opinions varied between sharing with either academic or pharmaceutical company researchers. Because a number of more dynamic and interactive consent approaches are being proposed to increase patient involvement (21), attention was paid as to how patients would like to exert control over sharing their samples and data.

## Materials and Methods

### Interviewees

Recruitment of cancer patients currently participating in a cancer clinical trial was undertaken at the gastroenterological or oncogynecological day hospital of the University hospital in Leuven, Belgium, through purposive sampling. All contacted participants took part in the study (*n* = 16). All participants were provided with an oral explanation of the study and a patient information sheet describing the study. Next, they were asked to sign an informed consent form (ICF) before the start of the interview. All patients had reached the age of majority. Patients with either gynecological or gastroenterological cancer were invited for the interview. The patients were at the UZ Leuven for their treatment at the time of the interview, so they did not have to make extra time for the interview.

### Interview Guide

An interview guide was developed based on available literature and was optimized by a team of experts active in the research field (Supplementary Material 1). The interview guide was piloted with non-cancer patients (*n* = 5) to ensure questions were drafted in lay language. The interview questions related to the following topics: (i) demographics; (ii) (re-)use of data and/or samples, (iii) use of data and/or samples by academia or industry, (iv) approval by ethics committee, (v) e-consent platform.

### Data Collection

The interviews (*n* = 16) were conducted face-to-face by three interviewers using the same interview guide in February 2017 and lasted about 30 min each. Recruitment ceased once data saturation was established. All interviews were conducted in Dutch. Written informed consent was obtained prior to the interview. The interviews were audio-recorded and transcribed *ad-verbatim*.

### Data Analysis

Interviews were pseudonymized and analyzed deductively via a content analysis by three researchers, based on the QUAGOL method (29). Interviews were coded and analyzed in Dutch. All concepts and codes were collected in writing and discussed orally amongst involved researchers. On such basis, consensus could be reached in all cases. The final text was translated in English after analysis.

## Results

Of the 16 participants, 9 (56%) were women. Ages ranged from 35 to 79 (mean 62, median 64). With the exception of one Polish woman, all participants described themselves as being Belgian. Participants had following cancer types: colorectal cancer (*n* = 4), ovarian cancer (*n* = 3), gastric and lung cancer (*n* = 1), colorectal and lung cancer (*n* = 1), pancreas cancer (*n* = 2), gastric cancer (*n* = 2), cholangiocarcinoma (*n* = 1), unreported (*n* = 2). Of the 16 participants, 10 participants reported to have followed higher education, of which six participants had completed college or university studies, and six participants did not enroll in any higher education.

### Sharing Data vs. Sharing Samples

Interviewed patients were aware of certain types of samples (e.g., blood, tumor tissue…) and data that are being collected. However, the majority of these patients did not seem to make a distinction in the sharing and re-use of their data vs. their samples. Moreover, interviewed patients reported only little interest about the purposes for which their data and samples are being used. They trust the clinicians to use the data and samples correctly in the scope of the research related to their disease. One stated for example:

“*I think they took a biopsy but I do not know much about it actually… but if it is in the context of the study, yes then I think it is normal that you give away these pieces.” (patient #6)*

And another:

“*We got a document stating what would happen (to our data and samples), which we approved without reading it in detail.” (patient #9)*

### Re-Use by the Same or a Different Research Team

All participants hypothetically allowed that their data and samples would be used by the original research team for further research, as long as, according to one participant, the research “*stays within the oncology research area*” (patient #2), again highlighting the level of trust in the initial research team. None of the participants found it necessary to be asked to re-consent in such case.

Participants appreciated medical research, and encouraged data re-use out of altruistic reasons, i.e., to help other or future patients as much as possible. Two patients even found it their duty to contribute to science, and expressed strong hopes that the maximal potential of their data and samples would be extracted:

“*It is only rarely that they find sufficient people to participate, so I feel that if you are eligible (for a study), that in some way it is your duty… because for instance, in my study now, we are only with seven patients.” (patient #2)*

“*If you can help other people, you have to help other people (…) so it would be better if everything would be more open and used.” (patient #6)*

Less than half of the interviewed patients indicated that they would like to be informed of any further use, “*if this would be possible*” one patient continued (patient #7). Of this group, some expressed a sense of curiosity, whereas others find it important that patients are informed about such further use because of transparency reasons:

“*I would like to be informed, definitely. In principle, I do not object to such further use, but I would like to have as much information as possible, so that at least I know what the research is about. Absolutely.” (patient #8)*

The majority of participants, however, did not find it necessary to be informed. One in particular described concerns about an abundance of unnecessary e-mails, which he perceived as annoying. Rather, he encouraged full “open use” of his donated data and/or samples (patient #12).

The desire for control seems to be greater in case of secondary use by a research team other than the original study team. At the one extreme, one patient favors complete open data re-use, thereby renouncing any form of control, on the condition however, that the secondary purposes are limited to research:

“*Everything is allowed by me. I would make data fully accessible. Of course, not for other purposes like advertisement; no, no, only for research purposes.” (patient #13)*

In contrast, two patients tended to distrust these unknown researchers and expressed concerns relating to misuse and security of their data.

“*I prefer to be asked (…), otherwise (researchers) can give away everything without informing anyone.” (patient #3)*

Overall, participants acknowledged the scientific value of re-use of their data and/or samples by another research team. Yet, in this case, some expressed a wish to be informed, again mostly out of curiosity reasons, i.e., to know in which studies their data are being used, by whom and to know to what they contributed. Patients would also like to be informed because this provides them with some form of verification on who is using their data; and thus, to ensure that there is no misuse of their data and samples.

“…*I would like to know what they would… What their plans are or…just out of curiosity” (patient #8)*

It should also be noted that two participants explicitly specified that the information provided to other research groups would be anonymized or coded, illustrating a wish to protect their privacy. If this can be secured, only little risk was perceived and thus the willingness to share increased.

“*Apparently everything happens coded, and as long as that is the case, I don't have anything against it.” (patient #5)*

### The Role of Ethics Committees

Further, we asked participants to consider the idea of an independent ethics committee (EC) that would decide about the re-use of samples and data for further research projects on their behalf. All but two interviewed patients liked this idea, stating that they have trust in the fact that these people will have a good level of expertise to make appropriate decisions, “*as long as there is just some form of control*” (patient #2). Some even felt more comfortable with an independently appointed body making such decisions for them, since such a body is more knowledgeable to do this.

“*Yes they can because with their education and everything, they will know what to do. … by the way…my education does not have any link with these things…so…what can my opinion contribute to what is happening? I understand very little of all of this… why should I even want to….” (patient #8)*

However, even if the EC makes the decision in their place, a number of patients very much insisted to be informed about the further research purposes:

“*I would trust a body like an ethics committee, but I insist: I would like to be informed, logically (…)” (patient #8)*

Two participants held contrary views on the intermediary of an independent ethics committee. They indicated to find such control unnecessary, favoring open use of their data and/or samples (patient #12 and #13).

### Opinion on For-Profit and Not-For-Profit Research

Subsequently, participants were asked whether their opinion about re-use would be different when it constitutes academic or pharmaceutical industry research. Two patients preferred their data to be shared and used by academic researchers rather than by pharmaceutical companies, with the simple reason that pharma companies have commercial interests.

“*Yes, this is different for me. I would prefer it to be a university, maybe because they are independent. Of course you can say “but you also entered a study, and it is a commercial study,” but yes you look after yourself, which is logic, but ideally it would be better if this would be a university, the research centers that are independent vis-à-vis such studies” (patient #7)*

Interestingly, the majority of patients did not make this distinction. Even if the goal of companies is to make profit, in the end, they achieve this by bringing treatments to the market and therefore, patient data and samples should be shared as much as possible.

“*No, I am not selective on this point, no. This is the same as…these are all people working for the same goal. Pharmaceutical companies are involved in research, because they make the medicines…” (patient #5)*

“*It all boils down to the same thing; for the company of course there is money involved but in the end it is for the patients” (patient #13)*

However, patients did deplore a lack of sharing of data and/or samples because of commercial reasons and some expressed that this protective attitude should not be allowed. Some interviewed patients expressed great hopes that researchers share and collaborate to exploit the full potential of the participants' data and samples.

“*They should bring together all these data, and aim to achieve goals together since in the end everybody is doing research for the same purpose (…). If you invent a coffee pot, I can understand that you want to protect your invention, but this is about human lives, the wellbeing of people.” (patient #2)*

While the majority expressed the view that scientific advances and medical research should be the greatest motivation, some understood that pharmaceutical companies are protective over the sharing of data and/or samples:

“*I can understand from a company's perspective that you want to protect those things, but if it could benefit other people… it would be better if they would open up the data.” (patient #6)*

One participant even clearly stated that the donated material belongs to the study sponsor, since they invested a lot of money in collecting it (patient #11). Therefore, this patient found it appropriate that it is up to the study sponsor to decide with whom he shares the data and/or material.

“*I can relate to that, the pharmaceutical sponsors have put a lot of capital in that, and you also have patents and so on… I think it is good that this (material and data) belongs to them and that they can determine either yes or no. In the end, this is their material and data” (patient #11)*

### Interactive, Electronic Tools for Increased Patient Control

Some participants have a desire for greater involvement and/or greater need for information. This was reflected when we introduced the idea of a more interactive consent tool where they could individualize their preferences toward their data and sample management. Interviewed patients were positive about the use of an electronic platform that provides opportunities to enable greater control over their consent. Participants highlighted that today's consent practices do not allow to indicate what can happen with the donated data/samples or how they would like to be informed about any further use or to get research results communicated back to them. Although the majority of interviewed patients mentioned that they would share their data openly without any further limitations, consent practices incorporating such preferences were found useful.

“*That would be really easy as a matter of fact. This does not exist yet and it would be really interesting for patients” (patient #6)*

Even though many interviewed patients indicated that it would not be of relevance to them (since they were not actively working with multi-media devices), they recognized the importance for other, more IT-minded people. Especially, some acknowledged such tools to be beneficial for those putting more emphasis on their privacy or their individual preferences. As a condition for use, however, the privacy and security of those systems should be guaranteed. Two participants clearly expressed concerns about multi-media devices replacing the personal doctor-patient contact, which is perceived as very valuable. Despite the potential benefits, one participant (patient #8) expressed his distrust against new, electronic systems. Although it was explained during the interview that such tools would not replace (but rather support) the personal doctor-patient contact, he feared electronic tools to become alternatives of the traditional care provision and treatment.

## Discussion

The current study presents the opinion of cancer patients participating in a clinical trial on a number of themes that may affect the willingness to share data and samples. These themes (the re-use by the same or a different research team, the role of independent ethics committees, the opinion on for-profit and not-for-profit research and the value of interactive, electronic tools for increased patient control) were introduced to the participants during face-to-face interviews. A number of key findings can be derived from our study that should be taken into account when designing patient-approved data/sample sharing frameworks in clinical research.

First, most of the cancer patients interviewed in this study have the view that their data and/or samples can and should be re-used to stimulate medical research in their disease domain. Participants felt that it is their duty to contribute to science, almost as if it is their social responsibility to do so. In this respect, the current results echo those by Jones et al. (28). However, our results indicate even more liberal attitudes toward data sharing. One reason might be that where Jones et al. targeted a broad patient population, we specifically targeted oncology patients participating in a clinical trial in the University Hospital of Leuven (Belgium). Considering their disease status and participation in a trial, it may be that our target group is more open toward sharing and re-using with the ultimate aim to support research; whereas a number of patients included in the study of Jones et al. are slightly more risk averse. The question may arise whether patients with cancer place a greater premium on the public benefits of medical research, and less on their individual rights to privacy. This is important, since overemphasizing such individual rights could present challenges to the conduct of activities performed for public rather than for individual benefit, for instance medical research. Or as Selinger puts it: “*Total autonomy of one individual can have a negative effect on autonomy of other individuals”* since one could approve data use for his own treatment, but hamper it to improve care for others (30).

Second, even though interviewed patients clearly want to contribute to advances in medical research, they showed little interest in the specific purposes for which their data and samples are being used. This finding is in line with the results from Mello et al. that showed that the willingness to share data is not really affected by the purpose for which the data would be used (31). It suggests a form of institutional trust in the hospital as well as in the clinicians, but this also raises questions about how well research participants read and understand ICFs. Moreover, this study indicates that trial participants view data and samples as similar resources, while from a legal perspective they are not considered the same, which complicates their re-use and/or sharing.

Third, although participants support re-use by the initial and other research teams, divergent opinions exist as to the level of control and patient involvement, which is in line with the results from two quantitative surveys by Shah et al. (32, 33). A small group of participants favored completely open use of their donated data and/or samples, thereby renouncing any form of control. These patients are comfortable as being “passive observers” of the whole research project. Considering the myriad of initiatives initiated to increase “patient empowerment,” “patient centricity,” and “patient engagement” the last few years (34), it is important not to obfuscate this finding: we should not overdue patient involvement or put an undue burden on patients to actively manage their care process where this is not desired. The majority of participants favored easy re-use but valued a higher degree of control/engagement in this process. However, it was recognized that the lack of opportunities for greater involvement complicates this. Lastly, another small group of patients strongly felt the need for being actively involved (i.e., by re-consenting) when data is shared with initially unknown research groups. Although these patients did not object to such sharing, they expressed concerns about security and a lack of trust with respect to potential recipients. Trust and transparency about data and sample sharing arrangements is of utmost importance in medical research since experience of inappropriate disclosure could negatively impact on participants' willingness to share information, or at worst, avoid future participation (4).

Fourth, all but two patients expressed their trust in ethics committees taking up the task of intermediary decision maker. In a previous quantitative study with 2,005 patients with rare diseases, about half of the respondents indicated that they would allow an ethics committee to decide on their behalf (35). Our finding reflects the practices as prescribed by ethical recommendations such as the Helsinki Declaration, although not echoed in all legal frameworks since the EU data protection framework does not stipulate any intermediary form of control for secondary re-use of sensitive data. In general, confusion exists among researchers about whether or not informed consent is needed for re-use of data for further research. The General Data Protection Regulation (GDPR) stipulates different legal grounds for processing of personal data. Aside from explicit consent from the participant [Art. 6(a)], public interest [Art.6(e)] may also be considered. The GDPR leaves it up to member states to define what constitutes “public interest”. Belgian law does not mention scientific research as a type of public interest. Therefore, consent for research may remain the important legal basis for re-use of personal data in Belgian context.

Fifth, patients in this study expressed only few concerns about the for-profit/not-for-profit nature of organizations, explaining that even if pharmaceutical companies are driven by profit, their profit is made by developing products that benefit patients, thus ultimately all medical research serves the same purpose. This finding is somewhat contrary to the results from previously published quantitative studies, which indicated that research participants and rare diseases patients were more likely or comfortable to allow their data to be shared with not-for-profit stakeholders (e.g., academic researchers, health care professionals, non-profit and patient organizations) than with researchers in for-profit companies or insurance companies (31, 32, 35, 36). Yet, most participants in our study did mention that they deplore a lack of collaboration and sharing between researchers because of commercial reasons.

Finally, digitalization has opened up new possibilities for patients to be engaged in research. However, beyond the current popular rhetoric of patient empowerment, this study aims to clarify patients' attitudes concerning the use of new tools to consent and to enable greater control over data and/or sample management. Participants were mostly positive about the use of such tools, and valued, besides increased control and transparency, the possibility for the provision of feedback from research results. Some patients explicitly recognized that even if privacy was less important to them, individualized consent methods could be valuable to others paying more attention to their privacy. However, there is an important issue to consider when thinking of implementation of e-consent tools. One should carefully consider the consequences when conducting research based on data from “information altruists,” especially the potential selection bias. Previous research reports that, from the general public, those with higher educational qualifications are more likely to share their EHRs (37). Further, it was recognized by almost all participants that in practice, such system might not yet be of direct benefit to them (which can be linked to the high age of the participants). However, they acknowledged such an approach to be more important for younger people or in the future. Nonetheless, technological (e.g., security), operational (e.g., ease of use), and legal concerns (e.g., privacy) were expressed. Importantly, interviewed patients highly valued personal contacts with their treating physician, emphasizing that in the existence of such system, this should not replace these face-to-face discussions.

Although, the current qualitative study provides some interesting new insights into different aspects that may affect a patient's (un)willingness to share his or her data and samples, it is exploratory in nature and has some important limitations. First, this is a single center study with a small sample size and a homogenous cohort (i.e., gastroenterological/oncogynecologic diseases only). Consequently, the study results are not generalizable to other patient groups or countries. Patients with a chronic or terminal illness might be more willing to share data in comparison to patients with better health outcomes, lower impact, or higher stigmatization. In addition, other factors influencing a patient's willingness to share could include culture, educational level or sociodemographic factors. Second, this study applied qualitative research methods only (i.e., in-depth interviews), so our results do not allow us to quantify the patients' perspectives and we cannot draw any conclusions about potential links between demographic parameters (i.e., disease stage, educational level, age, and sex) and the willingness to donate data and/or samples. A follow-up quantitative study, through surveys for example, could be useful to investigate this in more detail. Of note, a number of large-scale, quantitative studies investigating similar topics regarding data and/or sample sharing in various study populations have been published in recent years and should be taken into consideration when designing future research projects (31–33, 35, 36, 38). Third, the questions in Part III and IV of the interview guide refer to hypothetical situations, meaning that patients' answers may differ from real-life decisions. However, the patients in this study were in fact participating in a clinical trial so they could relate well to these situations. Fourth, only adult patients currently participating in a clinical trial were included. As a result, this study cannot draw conclusions about the need for re-consent to use samples and data of pediatric cancer patients once they become adults who can consent on their own behalf, which would be an interesting topic for additional research. Finally, additional themes were brought up by patients during the interviews, such as reciprocity (e.g., the need to communicate back research results). However, further research is needed to better understand these topics, which is why they were not discussed in more detail in this study.

## Conclusion

Discussions about clinical trial data sharing have largely taken place among experts. This study indicates a willingness of cancer patients participating in a trial to re-use their trial data and/or samples by the same research team, and a generally open approach to share these with other research teams albeit with the provision of information. Although the majority of interviewed patients had not thought much about sharing their data and/or samples in advance, they regretted the current lack of re-use and expressed wishes for (both for-profit and not-for-profit) organizations to collaborate in the future, to ensure the optimized use of their data and/or samples to achieve therapeutic improvements for fellow patients. Divergent opinions exist about how patients prefer to be engaged, ranging from passive donors to more actively involved patients, up to those explicitly wanting more control. To respect all attitudes, a stratified approach may be useful, in which those patients who want to have more say in the potential re-use of their donated data and/or samples can do so, for instance by e-consent approaches allowing individualization of preferences. However, the implementation of such an approach warrants further research and goes hand in hand with fully informing research participants about how their donations may be broadcasted and used by others. Educating and informing the patients sufficiently about the risks and the benefits of increased sharing is a sine qua non for participating more actively in the process.

## Data Availability Statement

The raw data supporting the conclusions of this article can be made available by the authors, without undue reservation, to any qualified researcher.

## Ethics Statement

This study protocol was approved by the Ethics Committee of UZ Leuven, Belgium (reference: S59829). All subjects gave written informed consent in accordance with the Declaration of Helsinki.

## Author Contributions

IH, DL, MC, and SB developed the idea for and were involved in the design of this study. SB and CV reviewed available data sources and drafted the manuscript. IH, DL, and MC critically revised the manuscript. All authors read and approved the final manuscript.

### Conflict of Interest

The authors declare that the research was conducted in the absence of any commercial or financial relationships that could be construed as a potential conflict of interest.
