# Supplementary material for: Sharing of Clinical Trial Data and Samples: The Cancer Patient Perspective
Source: Front Med (Lausanne). 2020 Feb 11;7:33. doi: 10.3389/fmed.2020.00033 (PMC7026937; doi:10.3389/fmed.2020.00033)
Supplement: Supplementary file 1 [file Table_1.DOCX]

**Supplementary Material 1 - Outline of the interview guide**

Part I - Opening question

1. Demographic information (age, education, disease)

Part II – General questions relating data and samples collected during the trial

1. Do you know which information and/or bodily material from you were collected?

Part III – Use of this data and/or samples

1. What is your opinion when these data and/or samples would be used for other, related scientific research by the same research team?
2. Do you make a distinction between data and samples in such case?
3. In such case, would you like to be informed on this?
4. In such case, would you like to be asked permission for this?
5. What is your opinion when these data and/or samples would be used for other, related scientific research by the a different research team?
6. Do you make a distinction between data and samples in such case?
7. In such case, would you like to be informed on this?
8. In such case, would you like to be asked permission for this?

Part IV – Various

1. Often, study sponsors can be very protective of the data and samples that they have collected during their research. This could prevent other researchers from being able to reuse these data and samples for other research purposes. What is your opinion on this?
2. Why or why wouldn’t you find it acceptable and reliable enough if an independent ethics committee would decide in your place if the data and samples are allowed to be reused in a related scientific research?
3. Why would or wouldn’t it make a difference to you if your data were to be shared with academic researchers, like for example universities or independent research groups, or with pharmaceutical companies?
4. Opinion regarding an electronic consent platform

Part V – Closing questions

1. Any further comments?
